# Prevalence and associated factors of eating disorder symptoms in adolescents: a cross-sectional school-based study

**DOI:** 10.1186/s12888-023-04898-3

**Published:** 2023-06-02

**Authors:** Roges Ghidini Dias, Ricardo Rodrigo Rech, Ricardo Halpern

**Affiliations:** 1 Federal University of Health Sciences of Porto Alegre; University of Passo Fundo, Porto Alegre/Passo Fundo, Brazil; 2 University of Caxias do Sul, Caxias do Sul, Brazil; 3 Federal University of Health Sciences of Porto Alegre, Porto Alegre, Brazil

**Keywords:** Adolescent, Feeding and eating disorder, Body image, Obesity, Overweight

## Abstract

**Purpose:**

To estimate the prevalence of eating disorder symptoms and associated factors in adolescents between 14 and 17 years old.

**Methods:**

The data were obtained from a cross-sectional school-based study with 782 adolescents from public schools in Caxias do Sul, in Rio Grande do Sul, Brazil, in 2016. The Eating Attitudes Test (EAT-26) was used to investigate eating disorder symptoms. The chi-square test and Poisson regression with robust variance were performed to estimate the prevalence ratios and associations between the outcome and the variables of interest.

**Results:**

The prevalence of eating disorder symptoms was around 56.9% among adolescents and more prevalent in females. A significant association was found between eating disorders and female gender, mothers who did not study or had an incomplete elementary school, and body image dissatisfaction. To adolescents dissatisfied with being overweight, the prevalence was more than three times higher than that observed among those who did not report such dissatisfaction.

**Conclusion:**

The presence of eating disorder symptoms was associated with female gender, maternal education, and body image dissatisfaction. The results show the need to identify early signs and symptoms related to changes in eating behavior and non-acceptance of their bodies in a population especially concerned with their physical appearance.

## Introduction

Adolescence is recognized as a critical moment for the establishment of healthy behavior throughout life [1] and is marked by rapid growth and physical, cognitive, social, emotional, and sexual [2]. The way adolescents perceive the changes or the phase of this phase interferes with their social life and mental health [2], and the occurrence of changes that cause self-running problems, depression, and eating disorders (ED) are common in adolescence [3].

ED symptoms are attitudes, in addition to unique behaviors related to food, including fear of weight gain, desire to lose weight from insufficient dietary intake, food compulsion, self-in vomiting, and use of ingestion, among others [4]. These behaviors are healthy and can be the development of an adolescent, in addition to the development of obesity in adulthood [4, 5].

The prevalence of ED symptoms is attributed to some psychosocial factors, such as body image dissatisfaction (BID), low self-esteem, gender, and depressive habits, and is also associated with socioeconomic factors, such as education level and family income [6]. A study carried out in Rio de Janeiro investigated the evolution of the prevalence of ED symptoms and identified that at the end of five years, the chances of adolescents developing ED increased by 1.95 (OR = 1.95; 95%CI1.97–5.3) to 7.02 (OR = 7.02; 95%CI 2.81–17.54), which is significantly higher for females [7]. Although the issue of gender exerts a strong influence on ED, risk factors such as bullying victimization, influence and use of medication also increase the risk of being associated with this project [8].

Previous literature [9] points out that the presence of obesity in pediatrics increases the risk of developing ED, especially bulimia nervosa and binge eating disorders, both characterized by atypical eating behaviors or weight control. Another study identified statistically significant associations between ED and low self-esteem, BID, and the use of social media [10]. The findings presented in this study suggest a relationship between fitness concerns, BID, and inappropriate eating attitudes, especially in girls. Argyrides et al. [11] observed that weight/physical appearance and dysphoria were the most significant risk factors for the development of ED for both genders, while the internalization of the thin ideal was significant only for girls. Such results reinforce that ED is part of the national and international reality.

This study aimed to estimate the prevalence of ED symptoms and to investigate the associated factors with this phenomenon in a population of adolescents in the city of Caxias do Sul. Caxias do Sul is one of the largest cities in southern Brazil, with a population of approximately half a million inhabitants. Considering that the adolescent population is not in the habit of seeking medical services or other health professionals, unless they are conducted by a guardian, this study sought to highlight some aspects related to the health of this historically neglected portion of the population. We hypothesized that: (i) the prevalence of ED in adolescents would be positively correlated with younger adolescents (14 to 15 years old), female, with overweight and BID, and; (ii) sociodemographic variables such as school location and maternal education would be positively correlated with ED symptoms.

## Methods

A cross-sectional school-based study was carried out with adolescents aged 14 to 17 years old, regularly enrolled in the final years of elementary school (7th, 8th, and 9th grades) in public schools in the urban area of Caxias do Sul - RS. The study is part of the research entitled “Obesity, Body Image Dissatisfaction and Symptoms for Eating Disorders in a Cohort of Schoolchildren from the Serra Gaucha” [12]. Details of the methodology used in the baseline study are available in the literature [12].

The sample composition by cluster was used, calculated by the Epi Info 7.2.5.0 program (Centers of Disease Control and Prevention, Atlanta, United States), considering the population of students enrolled in the municipal education network from Caxias do Sul – RS in the respective age group (N = 3180). To calculate the sample size, a prevalence of 22% was adopted based on the study by Ludewig et al. [13]. A confidence interval of 95% and a precision of 3% were adopted, and thus the minimum number of 616 adolescents was estimated. An effect size of 1.2 was adopted to avoid losses and refusals, totaling the number of 726 adolescents needed to carry out this study.

In order to avoid possible selection bias, twenty-two schools (clusters) were drawn to complete the minimum number of students to be evaluated, the sample being distributed proportionally according to the number of existing students, the size of the school, and the age of the adolescents. All students who met the eligibility criteria were invited to participate. The eligibility criteria for participation in the study were: being between 14 and 17 years old in the year of the research, being regularly enrolled in the education system and voluntary participation by signing the free and informed consent form.

Of the 798 adolescents who responded to the questionnaires, 782 were considered eligible to participate in the study, considering the adherence of students around 97.9% to the study. The others (n = 16; 2%) were excluded due a lack of information.

Data collection took place between April and May 2016 through the application of a self-administered questionnaire to adolescents, at a time determined by the schools participating in the research. The questionnaire consisted of six blocks of questions: 1) Identification data (age, gender, height, body mass; 2) Sociodemographic information (maternal education and school location); 3) Practice of physical activities and sedentary habits; 4) Body image; 5) ED symptoms and; 6) Bullying. Questions from blocks one to five were used in this research.

Body mass assessment was performed using a Plenna portable digital scale (accurate to 100 g) was used. Height was measured using a stadiometer (accurate to 0.1 cm) fixed and aligned with the base of the wall of the school collection room. During the measurement, the adolescents remained barefoot and with minimal clothing. The body mass index (BMI) was identified through the quotient of body mass by height in meters squared (Kg/m2) as recommended by Cole et al. [14] and classified according to the cutoff points proposed for the Brazilian population [15]. For this study, BMI results were categorized into overweight (overweight and obese) and not overweight (eutrophic). Underweight adolescents were not identified. All procedures for measuring height and body mass, as well as calculating BMI, were performed by two previously trained researchers.

The location of schools was classified into two categories (central area and peripheral area), being considered schools in the peripheral area, all those within a radius greater than or equal to two kilometers from the epicenter of the city of Caxias do Sul (coordinates 29°09’48” South and 51°10’46” West). Maternal education was identified through the question “Up to what grade did your mother study?” in the self-administered questionnaire, and this information was categorized into two categories (No or Incomplete Elementary School and Complete Elementary School). The practice of physical activities (yes or no) and sedentary habits (up to 2 h/day or more than 2 h/day) were measured using a self-administered questionnaire.

The BID and the respective classification were evaluated by the Children’s Figure Rating Scale [16]. The scale contains nine numbered silhouettes, with extremes of thinness and fat, with stable height, and is presented separately, according to gender, with positive values ​​indicating a desire for a smaller body (dissatisfaction with excess) and negative values ​​indicating a desire for a larger body (dissatisfaction with thinness). The use of the Children Figure Rating Scale in this study is based on previous research results. Adami et al. [17] demonstrated the first evidence of validity for a figure rating scale for Brazilian adolescents. The authors identified that individuals with higher BMI z scores chose larger body contours and also showed higher levels of body image dissatisfaction. Zitzmann and Warschburger [18] investigated the psychometric properties of the image rating scale in children and adolescents. The authors demonstrated that the figure evaluation scales reveal greater stability for ideal figure selections and body image dissatisfaction rates when using the scale with ascending order of figures.

The study outcome (prevalence of ED symptoms) was obtained through the application of the Eating Attitudes Test (EAT-26) validated for the population of Brazilian adolescents [19]. Those evaluated were classified according to the score obtained so that scores greater than or equal to 21 points were considered symptomatic and less than 21 points were considered asymptomatic.

Data were analyzed using the IBM-SPSS® version 23 program using descriptive and inferential statistics. The differences between the type of BID and the presence of ED symptoms were calculated according to the prevalence of students dissatisfied with their body image. A Poisson regression model with robust variance (backward stepwise method) was used to assess the factors that influenced the outcome, using each as a dependent variable separately, including only the variables that had p < 0.20 in the univariate analysis. The missing data of the variables of interest in this study were discarded, in order not to make the prediction model unfeasible. A confidence level of 95% and p < 0.05 were considered for all analyzes. This study was approved by the Research Ethics Committee of the Federal University of Health Sciences of Porto Alegre (UFCSPA) with opinion number 1312/11 and registration 741/11.

## Results

This study had the participation of 782 adolescent students. Of the total of 798 subjects able to participate in the study, a small sample loss (n = 16) was observed due to the free and informed consent form not having been signed by parents or guardians (n = 6) and lack of information about the variables of interest: no data for BID (n = 3), no data on maternal education (n = 6), and no data for ED symptoms (n = 9).

Table 1 shows the characterization of the sample according to the ED symptoms. The prevalence of ED symptoms was around 56.9%, being more prevalent in females (63.6%), aged between 14, and 15 years old (52.6%). Most participants who had ED symptoms studied in schools located in the central area of ​​the municipality (66.5%) and belong to families whose mothers did not study or have incomplete elementary school (70.6%).

**Table 1:** Comparison of variables of adolescents aged between 14 and 17 years by ED symptoms, in Caxias do Sul, 2016

Variables	Categories	ED Symptoms	
		Yes (n = 445)	No (n = 337)
Gender	Female	283 (63.6%)	79 (23.4%)
	Male	162 (36.4%)	258 (76.6%)
Age (years)	14 to 15	234 (52.5%)	180 (53.4%)
	16 to 17	211 (47.2%)	157 (46.6%)
Maternal Education	No or Incomplete Elementary School	314 (70.6%)	182 (54.0%)
	Complete Elementary School	131 (29.4%)	155 (46.0%)
School Location	Central	296 (66.5%)	238 (70.6%)
	Peripheral	149 (33.5%)	99 (29.4%)
Sedentary Habits	≤ 2 h/day	192 (43.1%)	168 (49.8%)
	> 2 h/day	253 (56.9%)	169 (50.2%)
Physical Activity	Yes	169 (38.0%)	127 (37.7%)
	No	276 (62.0%)	210 (62.3%)
BMI	No Overweight	244 (54.8%)	279 (82.8%)
	With Overweight	201 (45.2%)	58 (17.2%)
BID	Satisfied	159 (35.7%)	269 (79.8%)
	Not Satisfied	286 (64.3%)	68 (20.2%)
BID Classification^†^	With Excess	195 (55.1%)	-
	With Thinness	156 (44.9%)	-

BMI: Body Mass Index; ED: Eating Disorder; BID: Body Image Dissatisfaction; ^†^: n = 354; * Significant values at p < 0.05

It was observed that the majority of students who reported ED symptoms had longer daily sedentary habits (56.9%), did not practice regular physical activity (62.0%), were no overweight (54.8%), and dissatisfied with body image (64.3%). There was a predominance of dissatisfaction with excess (55.1%), denoting that most dissatisfied students wish to have a smaller body.

The crude analysis sought to show associations between the outcome and the predictive variables (age, gender, maternal education, school location, sedentary habits, physical activity, BMI, and BID). Confirm the Poisson regression assumptions with robust variance, only the variables gender, maternal education, BMI, and BID were submitted to an adjusted analysis. The other variables did not show a statistically significant relationship with the outcome.

In Table 2, on the crude analysis, the frequency of symptoms of ED on the part of students not overweight was more than twice as high as that of students with overweight. In the adjusted analysis, however, no such association was identified. It should be noted that the frequency of reference of ED symptoms by adolescents with BID was almost five times higher compared to students satisfied with their physical shape in the adjusted analysis (PR = 4.48; 95%CI: 2.75–10.8). A similar fact was observed in adolescents who wanted a smaller body (dissatisfaction with excess), who had more than three times the prevalence of ED symptoms compared to students satisfied with their body (PR = 3.73; 95%CI: 2.74–6.15). Furthermore, the results showed that the presence of ED symptoms was associated with female adolescents (PR = 1.33; 95%CI: 1.19–1.61) and whose mothers with no or incomplete elementary school (PR = 1.74; 95%CI: 1.46–2.05) .

**Table 2:** – Regression analysis between symptoms of eating disorders and independent variables in adolescents from public schools in Caxias do Sul,2016

Variables	Total	β	Wald	Crude Analysis		Adjusted Analysis ^†^				
	(n = 782)			PR (CI _95%_)	p-value	PR (CI _95%_)		p-value		
**BMI**										
No OW	523	0.142	0.024	2.36 (1.84–3.57)	0.034*	1.03 (0.82–1.27)		0.089		
With OW	259			1		1				
**BID**										
Not Satisfied	354	1.21	13.556	5.56 (3.02–9.81)	< 0.001*	4.48 (2.75–10.8)		< 0.001*		
Satisfied	428			1		1				
**BID Classification**										
By Excess	181	1.12	31.596	4.09 (2.28–5.26)	< 0.001*	3.73 (2.74–6.15)		< 0.001*		
By Thinness	173			1		1				
**Gender**										
Female	362	0.25	3.328	0.68 (0.51–1.02)	0.19	1.33 (1.19–1.61)		0.046*		
Male	420			1		1				
**Maternal Education**										
No or Incomplete Elementary School	589	-0.19	2.872	1.49 (1.18–1.66)	0.041*	1.74 (1.46–2.05)		0.043*		
Complete Elementary School	193			1		1				

^†^: Adjusted Analysis by Poisson regression with robust variance; n = 354; BMI: Body Mass Index; PR: Prevalence Ratio; CI: Confidence Interval; BID: Body Image Dissatisfaction; * significant values at p < 0.05

## Discussion

The present study, based on a representative school sample from one of the largest cities in southern Brazil, illustrates important data about a condition of extreme concern among the adolescent population. Overall, the results of our study shine a light on the high prevalence of ED symptoms, confirming that it is a very prevalent condition in Brazilian adolescents. Worldwide, the prevalence of ED tends to vary greatly due the cultural, racial, or ethnic differences and also due to the use of different assessment instruments, which may overestimate or underestimate these results [20]. The prevalence of ED is 9% in most parts of the world [21], and compared to international [21] and national [22] studies, the prevalence observed in our research was higher and causes great concern. Particularly when considering the results by gender, the rate observed among girls deserves to be highlighted (63.6%). The literature indicates that the onset of ED usually occurs during adolescence, and may be present in children and adolescents of both genders aged between five and 12 years, but with a higher prevalence in girls [23]. One of the factors that may be linked to the higher prevalence of ED among girls is the fact that they enter puberty earlier, experiencing physical changes and social pressures before boys. Adaptation to increasingly demanding esthetic standards such as thin bodies [11], the constant concern with weight gain, dieting without supervision [24], low self-esteem, depressive symptoms, and body dissatisfaction [6] are known factors that predispose the development of ED in young people, especially females. Considered worrying because of the numerous consequences that the BID can have among adolescents, the prevalence rate identified in our study can be explained due to the sensitivity of the instrument, since, due to the amplitude of the scale, the alteration of only one silhouette of difference is capable of classifying individuals as dissatisfied.

Leal et al. [22] found that adolescents who perceive themselves as overweight are almost twice as likely to acquire unhealthy eating habits, which favor the development of ED (OR = 1.79; 95%CI: 1.236–2.608). A recent study [25] reinforces these data, which were also observed in our research. Girls overestimate their body shape by desiring slimmer bodies, while boys and boys tend to underestimate their physical shape, desiring stronger and more athletic bodies. These conceptions may be related to the imposition of specific beauty standards for each gender [25], as well as the excessive exposure of young people to social media [26]. Distortions in the perception that adolescents have about their body image can potentiate a dangerous link between excessive preoccupation with appearance and harmful changes in eating behavior. The findings of our study reflect the internalization of a process of belief in thinness as an instrument for the acceptance and inclusion of adolescents in their social groups, reinforcing the belief that thin and slender subjects are more desirable [27]. Such results evoke the reflection that the concern to achieve a standard of beauty considered ideal is a phenomenon that is too present among young people and that has persisted between generations.

The literature points to positive associations between maternal education and adequate dietary practices. A study by Zadka et al. [28] identified a positive correlation between the frequency of consumption of vegetables (G = 0.167; p < 0.005) and fruits (G = 0.155; p < 0.005) and mothers’ education.

The literature points to a possible association between maternal educational level as an essential factor in triggering ED. Recent studies have identified that mothers with less food knowledge were more negligent and less responsible about to their daughters’ eating behavior [29]. On the other hand, adolescents with more educated mothers had a lower prevalence of unregulated eating behaviors and greater family support associated with nutritional issues.

Lawrence et al. [29] investigated a possible association between child health status and its influence on the maternal educational level in the US population between 2000 and 2017. The results showed that children whose mothers had less than eight years of schooling had worse health status (OR = 3.84; 95% CI: 3.62–4.07) about children whose mothers attended high school (OR = 2.57; 95% CI: 2.44–2.70) or if formed (OR = 1.90; 95% CI: 1.80–2.00), thus demonstrating a probable positive influence of maternal schooling on the evolution of the health status of the children under their responsibility. However, despite strong evidence, it is not possible to generalize these results.

Arendt et al. [30], when investigating possible benefits resulting from the increase in the duration of years of basic education in a generation of Danish students, were unable to identify a causal relationship between the increase in maternal education and its positive effects on the health status of their respective children. The authors explain that the results are because the reform mainly increased education at the lower end of the sample distribution, so greater effects of this variable on the health of children and adolescents would occur if there were changes in higher education levels. Another aspect that explains the results is based on the fact that the data come from a highly egalitarian country with a compressed income distribution and a large universal welfare state.

Although it is reasonable to believe that mothers with a higher educational level can encourage and lead their children to adopt healthy practices in relation to adequate food and that the increase in maternal schooling can act as a protective factor for the health of the child and adolescent population, it is necessary to adopt a cautious stance towards generalization. Our findings suggest highlight the importance of investigating EDs among adolescents, considering that the outcome is related to several other health events, which can generate serious consequences. ED is a current and worrying problem that has shown a strong association between female adolescents who had BID, and whose mothers studied for eight years or less. Although BMI is known as a predisposing factor for ED symptoms, the analyzes in our study showed no association of this variable with the outcome.

Some risk factors for the development of ED, although important, were not investigated in this study, so it is possible that this may impact the analysis of our results. The existence of traumatic and stressful events in the life histories of adolescents, such as sexual abuse in childhood [31], exposure to media that praise the culture of the ideal body, and also the identification of the social and family environment of adolescents can influence the way in which the results can be interpreted and understood as an even more complex and far-reaching phenomenon [32]. It is important to consider such statements in future studies, given that many factors are practically invisible and can be neglected [33].

Some limitations must be considered in the interpretation of these findings. First, although the number of participants is considered adequate in relation to the sample calculation, it is not possible to generalize the results of the study, considering that the sample was recruited in a single Brazilian city. Second, the study was cross-sectional, which precludes causal associations. Third, the sensitivity of the instruments used to assess BID and ED may have influenced the prevalence results among the investigated adolescents. Fourth, given that it was a cross-sectional study, there is a recall, and information bias among participants cannot be ruled out. Since the ED topic is still surrounded by taboos, there is a possibility that some participants have omitted some information. In addition, ED is influenced by multiple factors that were not analyzed in this work, thus requiring more detailed research.

On the other hand, these limitations were balanced by the strengths of the study. First, the sample gathered a significant number of participants, and their classification into two groups after the ED screening was relatively proportional, which guaranteed the power of the statistical analysis. Second, EDs were investigated using an instrument validated for the Brazilian adolescent population. Third, the methodological design allowed for controlling and reducing losses, reinforcing the findings of this study. Finally, the associations identified between the outcome and BID revealed worrying situations experienced by students and that should draw the attention of parents, health professionals, teachers, and students to the potential risks that ED and BID can trigger among adolescents.

It is of fundamental importance that there is dissemination and debate on the subject, as well as the elaboration of prevention and care strategies for adolescents involving the family, students, and the school community. Possible signs of changes in eating behavior should be identified and the matter should be dealt with transparently so that other problems related to the health of adolescents can be avoided. It is suggested that further studies be carried out with the follow-up of the dietary pattern of adolescents to elucidate possible relationships between the emergence of symptoms of ED and the consumption of certain foods.

## Data Availability

The data that support the findings of this study are available from the corresponding author (RGD) upon reasonable request.
